# Functional magnetic resonance imaging of the lumbosacral cord during a lower extremity motor task

**DOI:** 10.1162/imag_a_00227

**Published:** 2024-07-15

**Authors:** Christian W. Kündig, Jürgen Finsterbusch, Patrick Freund, Gergely David

**Affiliations:** Spinal Cord Injury Center, Balgrist University Hospital, University of Zürich, Zürich, Switzerland; Department of Systems Neuroscience, University Medical Center Hamburg-Eppendorf, Hamburg, Germany; Department of Neurophysics, Max Planck Institute for Human Cognitive and Brain Sciences, Leipzig, Germany; Wellcome Trust Centre for Human Neuroimaging, UCL Queen Square Institute of Neurology, University College London, London, United Kingdom

**Keywords:** humans, functional MRI, spinal cord, lumbosacral cord, motor activity, echo time

## Abstract

Blood-oxygen-level-dependent (BOLD) functional magnetic resonance imaging (fMRI) can be used to map neuronal function in the cervical cord, yet conclusive evidence supporting its applicability in the lumbosacral cord is still lacking. This study aimed to (i) demonstrate the feasibility of BOLD fMRI for indirectly mapping neural activity in the lumbosacral cord during a unilateral lower extremity motor task and (ii) investigate the impact of echo time (TE) on the BOLD effect size. Twelve healthy volunteers underwent BOLD fMRI using four reduced field-of-view single-shot gradient-echo echo planar imaging sequences, all with the same geometry but different TE values ranging from 20 to 42 ms. Each sequence was employed to acquire a single 6-min rest run and two 10-min task runs, which included alternating 15-s blocks of rest and unilateral ankle dorsi- and plantar flexion. We detected lateralized task-related BOLD activity at neurological levels L3-S2, centered at the ipsilateral (right) ventral spinal cord but also extending into the ipsilateral dorsal spinal cord. This pattern of activation is consistent with our current understanding of spinal cord organization, wherein lower motor neurons are located in the ventral gray matter horn, while interneurons neurons of the proprioceptive pathway, activated during the movement, are located in the dorsal horns and the intermediate gray matter. At the subject level, BOLD activity showed considerable variability but was lateralized in all participants. The highest BOLD effect size within the ipsilateral ventral spinal cord, as well as the highest split-half reliability, was observed at a TE of 42 ms. Sequences with a shorter TE (20 and 28 ms) also detected activity in the medioventral part of the spinal cord, likely representing large vein effects. In summary, our results demonstrate the feasibility of detecting task-related BOLD activity in the lumbosacral cord induced by voluntary lower limb movements. BOLD fMRI in the lumbosacral cord has significant implications for assessing motor function and its alterations in disease or after spinal cord injury.

## 1 Introduction

Functional MRI (fMRI) has been increasingly applied in the spinal cord to noninvasively map spinal cord neuronal function (Cohen-Adad, 2017;Haynes et al., 2023;Landelle et al., 2021;Powers et al., 2018). Spinal cord fMRI conducted in healthy volunteers has investigated imaging correlates of neural activity induced by a wide range of tasks and stimuli, including upper extremity motor task (Braaß et al., 2023;Govers et al., 2007;Hemmerling et al., 2023;Kinany et al., 2019;Stroman et al., 1999;Weber et al., 2016b), thermal stimulation (Bosma & Stroman, 2015;Rempe et al., 2015;Seifert et al., 2024;Weber et al., 2016a), tactile stimulation (Brooks et al., 2012;Kornelsen et al., 2013;Summers et al., 2010;Weber et al., 2020), noxious stimuli (Brooks et al., 2012;Summers et al., 2010;Tinnermann et al., 2021), and sexual arousal (Alexander et al., 2016;Kozyrev et al., 2012). Importantly, studies have reproduced the expected lateralization of spinal cord neural activity in response to ipsilateral motor tasks (Braaß et al., 2023;Hemmerling et al., 2023;Kinany et al., 2019;Weber et al., 2016b) or sensory stimulation (Brooks et al., 2012;Seifert et al., 2024;Weber et al., 2020), along with the expected rostrocaudal distribution (Kinany et al., 2019). Spinal cord fMRI has also been applied in the absence of explicit tasks (i.e., resting state) to uncover the intrinsic functional networks within the spinal cord (Barry et al., 2014,^2016^;Combes et al., 2023;Kinany et al., 2020;Kowalczyk et al., 2024;Vahdat et al., 2020;Weber et al., 2018). These networks are primarily characterized using data-driven approaches (Barry et al., 2016;Combes et al., 2023;Kinany et al., 2020,^2022^,^2024^;Landelle et al., 2023;Vahdat et al., 2020).

The majority of investigations thus far have focused on the cervical cord due to (i) the relatively high signal-to-noise ratio (SNR) facilitated by optimal coverage provided by the head and neck coils, (ii) the comparatively large cross-section of the cervical spinal cord, and (iii) the ease of minimizing task-related motion artifacts in the spinal cord during upper extremity motor tasks. In contrast, the lumbosacral cord, a region crucial for functions such as locomotion, reflexes, as well as sexual, bladder, and bowel control (Fowler et al., 2008;Krassioukov & Elliott, 2017;Sharrard, 1964), has received limited attention. This discrepancy can be attributed primarily to the smaller size of the lumbosacral cord and the technical challenges it presents, including the lower SNR associated with the spine coils and difficulties in shimming due to the proximity of large vertebrae and the lungs. Indeed, out of the 44 papers identified in a literature review (Landelle et al., 2021), only 1 focused on the lumbosacral cord. In that study, motor and sensory tasks were performed on six participants using a 1.5T MRI scanner, utilizing the signal enhancement by extravascular water proton (SEEP) contrast mechanism (Kornelsen & Stroman, 2004). In a recent study, blood-oxygen-level-dependent (BOLD) fMRI was employed to indirectly map neural activity in the lumbosacral cord during passive movements and muscle tendon vibration of the lower limbs in three patients with spinal cord injury (Rowald et al., 2022). Although yielding promising results, definitive evidence supporting the applicability of BOLD fMRI in the lumbosacral cord is still lacking.

A significant challenge in spinal cord fMRI is the lack of standardized imaging protocols, which limits the comparability of findings. While generic acquisition protocols and guidelines exist for quantitative MRI of the spinal cord (Cohen-Adad et al., 2021a,2021b), a comparable framework for spinal cord fMRI has yet to be established. Achieving this standardization necessitates a comprehensive understanding of how sequence parameters impact the fMRI results. In this context,Kinany et al. (2022)conducted a comparative analysis of frequently used fMRI sequences, evaluating their efficacy in detecting task-related activity and resting-state functional networks. However, their investigation focused on the cervical cord, which might not generalize to the lumbosacral cord. Therefore, there is a need to provide recommendations for optimal BOLD fMRI in the lumbosacral cord.

The goal of our study is twofold. First, we aimed to demonstrate the feasibility of lumbosacral BOLD fMRI by using a T2*-weighted single-shot gradient-echo echo planar imaging (GE-EPI) sequence during a unilateral lower extremity (ankle) motor task. Second, we investigated the influence of echo time (TE), an important determinant of BOLD sensitivity (Menon et al., 1993;Triantafyllou et al., 2011), on the level and extent of BOLD activity within the lumbosacral cord.

## 2 Methods

### 2.1 Participants

Twelve healthy participants (4 females, 8 males, age (mean ± standard deviation (SD)): 28.4 ± 3.4 years) participated in the study. All participants were classified as right footed based on their preference when kicking a ball to hit a target, picking up a pebble with the toes, stepping on a cigarette stump, and stepping up onto a chair (Tran et al., 2014). The study received approval from the Cantonal Ethics Committee of Zürich (BASEC ID: 2022-00558), and written informed consent was obtained from all participants.

### 2.2 Image acquisition

#### 2.2.1 Hardware and subject positioning

The scanning was performed using a 3T whole-body MR system (Magnetom Prisma, Siemens Healthineers) with a body transmit coil for excitation and a 32-channel spine matrix coil for reception. A foam wedge was placed under the legs to reduce the natural lordotic curve and maximize contact with the spine matrix coil. To minimize any movement of the lower spine due to the ankle motion task or involuntary motion, we (i) placed a vacuum cushion under the legs, (ii) applied body straps around the knees and hips, and (iii) instructed the participants to avoid heavy breathing. The ankle positioning allowed participants to perform the full range of ankle dorsi- and plantar flexion without touching the foam wedge.

#### 2.2.2 Structural scans

**Fig. 1.**
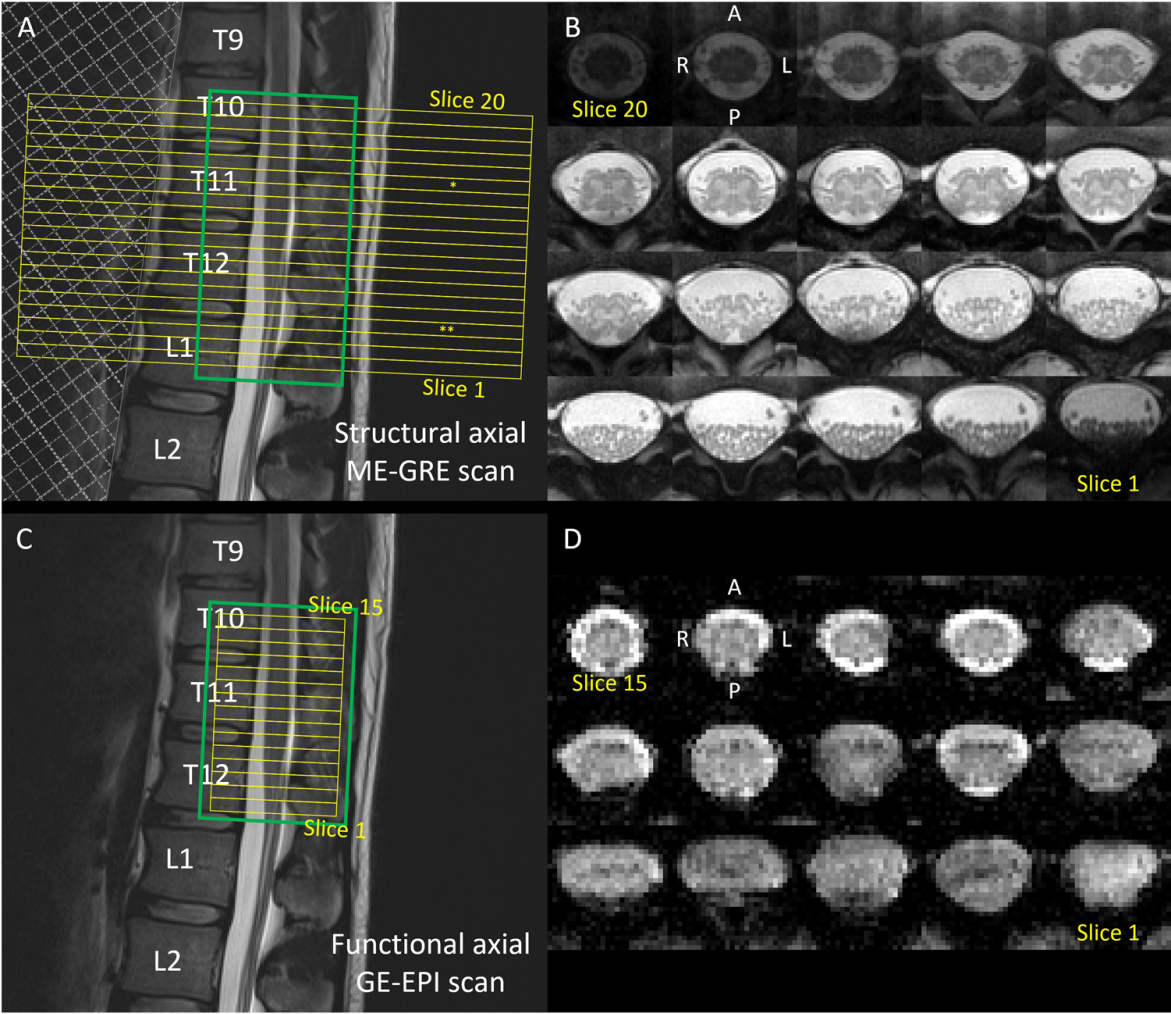
Slice positioning and example slices. Slice prescription was based on a sagittal T2-weighted turbo spin echo scan of the lower spine, for the (A) structural 3D spoiled multiecho gradient-echo (ME-GRE, Siemens FLASH) sequence and the (C) functional reduced field-of-view single-shot gradient-echo echo planar imaging (GE-EPI) sequences. The corresponding axial slices are shown for the (B) ME-GRE and (D) GE-EPI sequences (here, the iFOV35 version, seeTable 1), displayed in rostral (top left) to caudal (bottom right) direction. The slice stacks (20 slices for ME-GRE, 15 slices for GE-EPI) and shimming volumes are indicated by yellow and green boxes, respectively. The field of view (FOV) for the GE-EPI scan was set such that its sixth most rostral slice (slice #10) aligned with the maximum width of the spinal cord in the lumbosacral enlargement as observed in the sagittal T2-weighted image. The FOV for the ME-GRE scan was positioned to align the top edge with that of the GE-EPI scan (i.e., slice #20 in ME-GRE corresponds to slice #15 in GE-EPI), which ensured coverage of the lumbosacral enlargement and the conus medullaris. * indicates the slice with the largest spinal cord cross-sectional area (the “LSE landmark”) and ** indicates the most caudal slice of the spinal cord (tip of the spinal cord). For the ME-GRE sequence (A), a saturation band, indicated by a shaded area, was placed anterior to the spinal column to suppress potential artifacts originating from the abdomen. Note that the OVS20 version of the GE-EPI sequence (Table 1) also included two saturation bands, placed anterior and posterior to the FOV.

The substantial mismatch between vertebral and neurological levels in the lumbosacral cord (Canbay et al., 2014) precludes the use of vertebral levels as neuroanatomical landmarks. Instead, the lumbosacral enlargement (LSE) and the tip of the conus medullaris were suggested as reliable neuroanatomical landmarks (Büeler et al., 2024;Yiannakas et al., 2014). To facilitate the identification of these landmarks and the conus medullaris (the cone-shaped most caudal part of the spinal cord), a sagittal T2-weighted turbo spin echo sequence with 15 slices of 4.0 mm thickness (10% slice gap) was acquired (Fig. 1A, C). Additional sequence parameters were in-plane field of view (FOV) of 330 × 330 mm^2^, in-plane resolution of 0.7 × 0.7 mm^2^, repetition time (TR) of 3000 ms, echo time (TE) of 89 ms, echo spacing of 8.94 ms, turbo factor of 16, flip angle (FA) of 151°, phase oversampling of 75%, parallel imaging with GRAPPA (acceleration factor of 2), bandwidth of 272 Hz/pixel, and acquisition time of 00:59 min.

A high-resolution axial scan was acquired using a 3D spoiled multiecho gradient-echo (ME-GRE) sequence with 20 axial-oblique slices of 5.0 mm thickness (no gap) to serve as an anatomical reference (Fig. 1A, B) for the functional scans (Fig. 1C, D). Further acquisition parameters were in-plane FOV of 192 x 192 mm^2^, in-plane resolution of 0.5 x 0.5 mm^2^, TR of 38 ms, first TE of 6.85 ms, echo spacing of 4 ms, echo train length of 5 with bipolar readout, FA of 8°, phase encoding along the anterior-posterior direction, parallel imaging with GRAPPA (acceleration factor of 2), water excitation to avoid fat signal, flow compensation, bandwidth of 260 Hz/pixel, 6 repetitions, and acquisition time of 13:28 min.

#### 2.2.3 Functional scans

Functional scans comprised four T2*-weighted reduced FOV single-shot GE-EPI sequences with different TE, acquired with identical geometry. The 15 axial-oblique slices of 5.0 mm thickness were acquired in an ascending interleaved order without gap. While theoretically possible to vary only TE and investigate the effect of TE alone, we chose to adjust TR, FA, partial Fourier factor (pF), and the method of achieving reduced FOV (outer volume suppression (OVS) or inner field-of-view excitation (Finsterbusch, 2013)) for each TE (refer toTable 1for sequence parameters). The number of volumes was also varied to keep the acquisition time constant for each sequence. These adaptions allowed us to use optimal settings for each sequence and ensure a fair comparison between them.

**Table 1.** Sequence parameters of the single-shot gradient-echo echo planar imaging sequences used for functional MRI.

Sequence	OVS20	iFOV28	iFOV35	iFOV42
*Varying parameters*				
Type of reduced FOV	OVS	iFOV	iFOV	iFOV
Partial Fourier factor	6/8	6/8	7/8	no
Echo time (ms)	20	28	35	42
Repetition time (ms)	1460	1190	1290	1400
Flip angle (°)	77	72	74	76
Number of volumes (task)	411	505	466	429
Number of volumes (rest)	247	303	280	258
*Identical parameters*				
Number of slices	15			
Slice thickness (mm)	5.0			
Slice acquisition	interleaved			
FOV (mm ^2^ ) read x phase (L-R x A-P)	144 x 48			
Resolution (mm ^2^ )	1.0 x 1.0			
Echo spacing (ms)	0.93			
PAT	no			
Bandwidth (Hz/pixel)	1240			
Acquisition time (task)	10:05 – 10:07 min			
Acquisition time (rest)	06:05 – 06:07 min			

A-P, anterior-posterior direction; FOV, field-of-view; iFOV, inner field-of-view acquisition; L-R, left-right direction; OVS, outer volume suppression; PAT, parallel acquisition technique.

Three inner FOV GE-EPI sequences were acquired using Siemens’ ZOOMit implementation with varying TE (hereafter: iFOV28, iFOV35, and iFOV42, where the numbers at the end indicate the TE in ms). Shorter TE was achieved by applying pF in the phase-encoding direction, where TE was always set to the minimum (iFOV28: pF of 6/8; iFOV35: pF of 7/8; iFOV42: no pF). In addition, a single OVS EPI sequence (hereafter: OVS20) was acquired by placing two saturation bands, each with a thickness of 130 mm, anterior and posterior to the FOV, which allowed for an even shorter TE of 20 ms in combination with pF of 6/8. For each sequence, TR was set to the minimum and FA was set to the Ernst angle, using the given TR and a T1 estimate of 994 ms within the cervical gray matter at 3T (Smith et al., 2008). The only exception was OVS20, where the TR was increased from the minimum of 1130 ms to 1460 ms to comply with the specific absorption rate limit. All other sequence parameters were identical between the four sequences, including the in-plane FOV of 144 x 48 mm^2^, in-plane resolution of 1.0 x 1.0 mm^2^, phase encoding along the anterior-posterior direction, phase oversampling of 25%, and bandwidth of 1240 Hz/pixel.

### 2.3 Experimental paradigm and study design

**Fig. 2.**
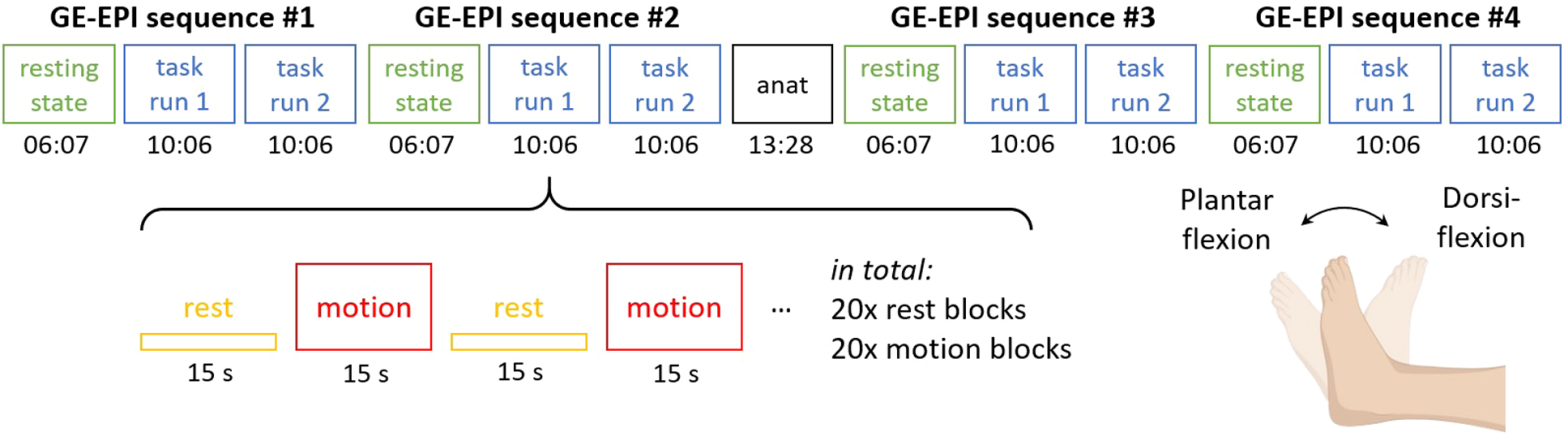
Experimental design and task paradigm. The experiment comprised four GE-EPI sequences, acquired in a counterbalanced order, with a structural scan acquired between the second and third GE-EPI sequences. Each GE-EPI sequence was used to acquire a single resting-state run and two task runs. The task runs consisted of alternating blocks of rest and unilateral (right-sided) ankle dorsiflexion/plantar flexion executed at a frequency of 0.5 Hz, that is, participants transitioned from dorsiflexion to plantar flexion and back in 2 s. Each block lasted for 15 s, with 20 motion and 20 rest blocks in each task run. Part of the figure was created withBioRender.com.

Figure 2illustrates the experimental paradigm and study design. The four GE-EPI sequences were acquired in a counterbalanced order. The structural scan was acquired between the second and the third GE-EPI sequence. For each GE-EPI sequence, we acquired one resting-state run followed by two task runs. The task paradigm consisted of alternating blocks of rest and right-sided ankle dorsiflexion/plantar flexion movements. The right side was chosen as it was the dominant side in all participants. Each block was 15 s long, and there were 40 blocks (20 motion and 20 rest blocks) in a task run, starting with a rest block.

During the task runs, participants observed the screen for instructions via a mounted mirror. A blank screen indicated rest (i.e., no ankle movement), while a flashing circle indicated ankle movement. Participants were instructed to perform the full range of dorsiflexion and plantar flexion at a constant rhythm according to the frequency of the flashing (1 Hz), meaning that participants transitioned from dorsiflexion (i.e., ankle in the highest position) to plantar flexion (i.e., ankle in the lowest position) in 1 s, and vice versa. During the rest runs, participants were instructed to lay still with their eyes open.

The task of ankle plantar and dorsiflexion was chosen for two reasons. First, we found that this movement can be performed without affecting the hip or lower back when proper subject positioning is applied (as described inSection 2.2.1). Second, this task is expected to induce activity at neurological levels L4-S1 (Rupp et al., 2021). These levels are situated at a distance from the lungs but where the spinal cord cross-sectional area is still reasonably large. Motor tasks that induce activity more rostrally or caudally might be harder to detect due to increased respiratory artifacts and the smaller size of the spinal cord, respectively.

Note that the identical acquisition times but different TR resulted in different number of volumes for both task fMRI (OVS20: 411; iFOV28: 505; iFOV35: 466; iFOV42: 429 volumes) and resting-state fMRI (OVS20: 247; iFOV28: 303; iFOV35: 280; iFOV42: 258 volumes) (Table 1). Three dummy volumes were acquired at the start of each GE-EPI sequence, which were discarded by the scanner and did not trigger the task paradigm. The total duration of the scanning session was approximately 2 h 10 min.

### 2.4 Processing of the structural images

The ME-GRE sequence yielded 30 images corresponding to 5 echoes in each of the 6 repetitions. In each repetition, we averaged all echoes into a single image (“combined echo”) by root-mean-square summation, followed by averaging across the six repetitions using serial longitudinal registration (part of SPM12) to account for motion between repetitions. To match the FOV of the cropped GE-EPI volumes, the resulting image was cropped in-plane around the center of the image to a matrix size of 97 x 97. The cropped image was used for further analyses and is referred to as the ME-GRE image throughout the paper. The ME-GRE image was segmented manually for spinal cord using the subvoxel segmentation tools in JIM (v.7.0, Xinapse Systems Ltd, UK). Subvoxel segmentations were binarized with an inclusion threshold of 50%.

### 2.5 Processing of the functional images

#### 2.5.1 Motion correction

The GE-EPI volumes were cropped in-plane around the spinal cord from a matrix size of 144 x 48 to 48 x 48 to reduce computing time and storage space. The GE-EPI volumes were motion corrected in a two-step process involving both volume- and slice-wise registration, similar to methods used previously (Weber et al., 2016a,2016b,^2020^). We adopted this approach because we observed that using slice-wise registration alone occasionally fails to correct for large slice displacements. First, we ran a volume-wise motion correction with rigid-body (six degrees of freedom (DOF)) transformation using MCFLIRT, applied with least squares cost function and spline final interpolation (Jenkinson et al., 2002) to register each volume to the mean EPI volume. The spinal cord centerline was generated on the mean motion-corrected EPI volume using the*sct_get_centerline*function in Spinal Cord Toolbox (SCT, v.6.2) (De Leener et al., 2017). Volume-wise registration was applied with a rigid-body transformation because we observed that superior slices, located closer to the lungs, tended to exhibit larger translations along the y-direction due to more intense breathing-induced fluctuations in the magnetic field. This slice-dependent y-translation, with more translation in the superior slices and less in the inferior slices, mimicked a rotation around the x-axis. Partial volume effects during interpolation were limited because the lumbosacral cord was relatively straight in all participants when using the subject positioning described inSection 2.2.1(seeFig. 1for an example), and estimated between-slice movements were small. Then, we fed the output into a regularized slice-wise motion correction algorithm (*sct_fmri_moco*), implemented in SCT and applied with two DOF (translation along x and y), first-order polynomial regularization function (along the slice direction), mutual information cost function, and spline final interpolation. To exclude areas outside the spinal canal which may move independently, the estimation of slice-wise motion parameters was restricted to a cylindric mask with a radius of 20 mm around the spinal cord centerline, created using the*sct_create_mask*function.

In the motion-corrected dataset, large intensity variations within the spinal cord between neighboring volumes were identified based on the DVARS metric (the spatial root-mean-square of the intensity difference image between successive volumes and within the spinal cord mask), as defined inPower et al. (2012)and computed using FSL’s (v.6.0.7.7) outlier detection tool (*fsl_motion_outliers*). Volumes with a DVARS exceeding the 75th percentile + 1.5 times the interquartile range were considered outliers.

#### 2.5.2 Segmentation

After motion correction, the mean motion-corrected EPI images were manually segmented for spinal cord in FSLeyes. The segmentation of the lower resolution and lower quality EPI image was guided by the segmentation of the higher resolution and higher quality ME-GRE image.

#### 2.5.3 Physiological noise modeling

Physiological noise regressors were obtained using the aCompCor method (Behzadi et al., 2007), implemented in the PhysIO toolbox (Kasper et al., 2017). The noise region of interest (ROI) was defined by manually segmenting the cerebrospinal fluid (CSF) in each slice. Special care was taken to exclude the interface between the CSF and the spinal cord from the segmentation to avoid partial volume effects, as well as the posterior part of the spinal canal due to the large number of nerves. No noise ROI was defined in the white matter due to the risk of partial volume effects and signal mixing with the gray matter. The underlying assumptions for noise modeling in the CSF are that (i) CSF does not contain signal of interest (given the absence of neurons) and (ii) the physiological noise in the CSF shares common features with that in the gray matter. For each slice, aCompCor extracted principal components from the fMRI data within the CSF ROI, ordered by their explained variance. Similar toBehzadi et al. (2007)andCombes et al. (2023), the first five principal components were retained.

#### 2.5.4 Coregistration to structural image

For each run, the mean motion-corrected EPI image was coregistered to the corresponding ME-GRE image using a nonlinear slice-wise registration method in SCT (*sct_register_multimodal*). The coregistration was based on the spinal cord segmentation of both images, given the low contrast, and was conducted in two steps: first, using the center mass transformation (smooth factor of 3 mm), followed by the BSplineSyn transformation (Tustison & Avants, 2013) (10 iterations, mean squares cost function, and spline final interpolation). Note that the warping field was not applied to the EPI images directly; instead, it was applied to the parameter estimates during the subject-level analyses.

#### 2.5.5 Normalization to template

The spinal cord mask of the ME-GRE image was spatially normalized to the PAM50 spinal cord template using the*sct_register_to_template*function (De Leener et al., 2018) to obtain the forward (native to template space) warping field and the backward (template to native space) warping fields. We employed a two-label normalization approach, where we assigned labels to the slice with the largest spinal cord cross-sectional area within the lumbosacral enlargement (referred to as the “LSE landmark”) and the most caudal slice of the spinal cord (tip of the spinal cord) in the ME-GRE image. These landmarks were matched with corresponding labels in the PAM50 space. Specifically, the tip of the spinal cord was aligned with “label 60” of the PAM50 template, and the LSE landmark was aligned with a manually added label (“label 59”) positioned at slice 143 (z = -490.34 mm). This slice corresponds to the middle of vertebral level T12 and the caudal end of neurological level L3, according to the PAM50 neurological segments based onFrostell et al. (2016). The forward warping fields were used in the group-level analyses to warp statistical parametric maps from the native to the PAM50 space. We also applied the forward warping fields to the structural ME-GRE and functional GE-EPI images to assess the quality of normalization.Supplementary Figure 1displays the normalized ME-GRE and GE-EPI images, averaged across all participants, as well as for a representative participant. These normalized images closely matched the PAM50 template for all participants.

### 2.6 Image analysis and statistics

#### 2.6.1 Temporal signal-to-noise ratio and outliers

The temporal signal-to-noise ratio (tSNR) is a measure of the temporal stability of voxel time series within the EPI dataset. tSNR is commonly used to assess the quality of fMRI acquisitions as it is related to the minimal detectable effect size in the BOLD signal (Murphy et al., 2007). For each GE-EPI sequence, voxel-wise tSNR was computed on the processed EPI images (cropped and motion corrected, as described inSection 2.5.1), which further underwent linear detrending (i.e., first-order polynomial detrending) using the Matlab function*detrend*. To calculate tSNR, the mean of the voxel time series was divided by its standard deviation using the*sct_fmri_compute_tsnr*function in SCT. tSNR was only computed for the resting-state runs, as task runs may contain BOLD-related signal fluctuations. A map of tSNR, normalized to the PAM50 space and averaged across all participants, is provided inSupplementary Figure 2. Subsequently, the tSNR values were averaged within the binary spinal cord mask in the native space, eroded by one voxel in the axial plane, to obtain a single tSNR value per slice. For slice-wise group statistics, axial slice stacks of individual subjects were aligned at their respective LSE landmark, that is, the slice with the largest spinal cord cross-sectional area. Differences in tSNR across GE-EPI sequences and slices were assessed using two-way repeated measures ANOVA, with sequence and slice as within-subject variables. An interaction term between sequence and slice was not included, as we did not anticipate any influence of slice on sequence differences. Post-hoc tests with Tukey’s correction for multiple comparisons (p < 0.05) were conducted to investigate pairwise differences.

The percentage of outlier volumes, as detected by*fsl_motion_outliers*, was computed for each task run. Any runs with an outlier rate exceeding 20% were excluded, ensuring that at least 8 min of data were available for each run-level analysis. A linear mixed-effects model, as implemented in the*nlme*library (v.3.1-164), was used to analyze differences in the rate of outliers between sequence types (OVS20, iFOV28, iFOV35, iFOV42) and sequence positions within the experiment (1st, 2nd, 3rd, 4th scan). Fixed effects included the sequence type and sequence position (within-subject factors), while participants were treated as random effects. Post-hoc tests, employing Tukey’s correction for multiple comparisons, were conducted using the*emmeans*library (v.1.10.1) to assess pairwise differences between sequence types and sequence positions. Statistical significance was set to p < 0.05.

#### 2.6.2 General linear model for task-based analysis

##### 2.6.2.1 Run-level analysis

For each of the four GE-EPI sequences (Table 1), the processed EPI images of the task runs were spatially smoothed using an anisotropic 3D Gaussian kernel with a full-width-at-half-maximum of 1 x 1 x 5 mm^3^. In the run-level (first-level) analysis of these images, a general linear model (GLM) was fitted on each voxel time series using FSL fMRI Expert Analysis Tool (FEAT) (Woolrich et al., 2001). The GLM design matrix included three explanatory variables and several nuisance variables. The explanatory variables comprised a boxcar function describing the motion task (0 for rest, 1 for motion), which was convolved with three basis functions generated by FMRIB’s Linear Optimal Basis Sets (FLOBS) toolkit (Woolrich et al., 2004). Nuisance regressors included two motion parameters (x and y translational parameters output by*sct_fmri_moco*), motion outliers (output by*fsl_motion_outliers*), and the five principal components extracted from the CSF. The motion parameters and principal components served as slice-wise regressors (i.e., they varied from slice to slice) to account for variations in noise along the rostrocaudal direction. Besides model fitting, FSL FEAT also performed high-pass temporal filtering (cutoff frequency of 100 s) and prewhitening (FSL FILM) on the voxel time series. As the rest block was not explicitly modeled, the contrast of parameter estimates (COPE) for the comparison (task > rest) corresponded to the parameter estimate of the first explanatory variable (β_1_).

##### 2.6.2.2 Subject-level analysis

The two COPE maps from the run-level analyses (runs 1 and 2) were warped to the ME-GRE image by applying the warping field obtained during coregistration (Section 2.5.4). The warped COPE maps were passed to the subject-level (second-level) analysis, which generated a subject-specific COPE map using a fixed effects model.

##### 2.6.2.3 Group-level analysis

The subject-specific COPE maps were warped to the PAM50 space using the deformation field obtained during normalization (Section 2.5.5). Group-level (third-level) analysis was done by performing a nonparametric permutation test using FSL*randomise*with the default variance smoothing of 5 mm (Winkler et al., 2014). Notably, nonparametric tests generate the null distribution instead of modeling it, which results in exact inference (Eklund et al., 2016). In each permutation, the algorithm randomly flipped the signs of the subject-specific COPE values (n = 12, resulting in 2^12^= 4096 possible permutations), and performed a one-sample*t*-test on the sign-flipped COPE values. On a voxel-by-voxel basis, the actual*t*-score was then tested against the distribution of the 4096*t*-scores, yielding an (uncorrected) p-value. Threshold Free Cluster Enhancement (TFCE) was applied on the uncorrected maps of p-values to enhance cluster-like features in a threshold-free manner (without a cluster-defining threshold). The TFCE p-maps were family-wise error (FWE) corrected using three thresholds: (i) p_FWE_< 0.05, following common thresholding practices, (ii) a more conservative threshold of p_FWE_< 0.01 to emphasize differences in activity maps across the four sequences, and (iii) an even more conservative threshold of p_FWE_< 0.001 to highlight only the voxels with the strongest association with the paradigm.

Three additional participants (one female, two males, age (mean ± SD): 29.8 ± 3.1 years) were recruited as controls to evaluate the false positive rate of activity. These participants underwent the same scanning procedures but were instructed not to perform the task during the task runs. The control datasets were processed and analyzed in the same manner as described above.

#### 2.6.3 Distribution of BOLD activity

In the cross-section, the spinal cord was segmented into five ROIs: right ventral (RV), right dorsal (RD), left ventral (LV), left dorsal (LD), and medioventral (MV) spinal cord (Fig. 6A). The ROIs were created manually based on the PAM50 spinal cord atlas. The RV, RD, LV, and LD ROIs were delineated with a one-voxel gap between them by excluding all voxels with x-coordinates of 70 or y-coordinates of 70 (voxel coordinates). The MV ROI was created to separate the anterior median fissure, which contains the sulcal vein, a large vein draining the ventral gray matter horns, from the RV and LV ROIs. This was achieved by drawing an inverse pyramid shape to include the anterior median fissure and surrounding areas while excluding voxels near the ventral gray matter horns (Fig. 6A). Along the rostrocaudal axis, the lumbosacral cord was segmented into approximate neurological levels using the PAM50 template, which defines these levels based on the previously described relative positions between neurological and vertebral levels (Frostell et al., 2016) (Fig. 6B). To quantify BOLD activity, the mean*t*-score and the ratio of significant voxels (p_FWE_< 0.01) were calculated within (i) each ROI over neurological levels L3-S2 and (ii) each neurological level over the whole spinal cord cross-section.

#### 2.6.4 BOLD effect size

The BOLD effect size was calculated as the percentage difference between the average signal intensities during motion and rest blocks within the task runs, for each participant and sequence. For each run, we generated the fully denoised dataset from the output of FSL FEAT. In particular, we summed up three 4D files: (i) the task-related signal, computed as the coefficient map associated with the first explanatory variable (task,$\beta_{1}$, stored in the*pe1.nii.gz*file) multiplied with the hemodynamic response function (HRF) (stored in the*design.mat*file), (ii) the offset signal intensity (stored in the*mean_func.nii.gz*file), and (iii) the residual errors (stored in the*res4d.nii.gz*file). Next, the mean time series was extracted within the RV ROI over the neurological levels L3 to S2, where motor-induced BOLD activity is expected from neuroanatomical considerations. For that, the RV ROI was transformed from the PAM50 to the native space using the backward warping fields obtained during coregistration and normalization. The mean time series were split into motion and rest blocks, where the first three data points of each block were omitted to exclude transient effects of the BOLD signal. Finally, we calculated the percentage difference between the mean signal intensities during motion and rest blocks.

#### 2.6.5 Reproducibility

Reproducibility of the group-level statistical parametric maps was evaluated through split-half reliability analysis for each sequence. The two COPE maps from the run-level analyses (run 1 and 2) were warped to the ME-GRE image, as described inSection 2.6.2.2, but were not combined into a subject-specific COPE map. Instead, two separate group-level analyses were run using solely the data from either run 1 or run 2. Similarity between the resulting two sets of TFCE p-maps was assessed by computing the Dice coefficient after binarizing them at various thresholds (p_FWE_< 0.05, p_FWE_< 0.01, and p_FWE_< 0.001).

## 3 Results

### 3.1 Temporal signal-to-noise ratio and outliers

Example slices of each GE-EPI sequence are provided inFigure 3A. As expected, images acquired with a shorter TE display higher intensity levels. In addition, images with lower partial Fourier factor appear smoother (6/8 for OVS20 and iFOV28, 7/8 for iFOV35, 1 for iFOV42). In all participants, the OVS20 sequence produced folder-over artifacts at the anterior edge of the images despite the use of saturation bands (see green arrows inFig. 3A). Occasional signal dropouts at the dorsal edge of the spinal cord, induced by susceptibility artifacts, were more prominent at longer TE (see blue arrow inFig. 3A).

**Fig. 3.**
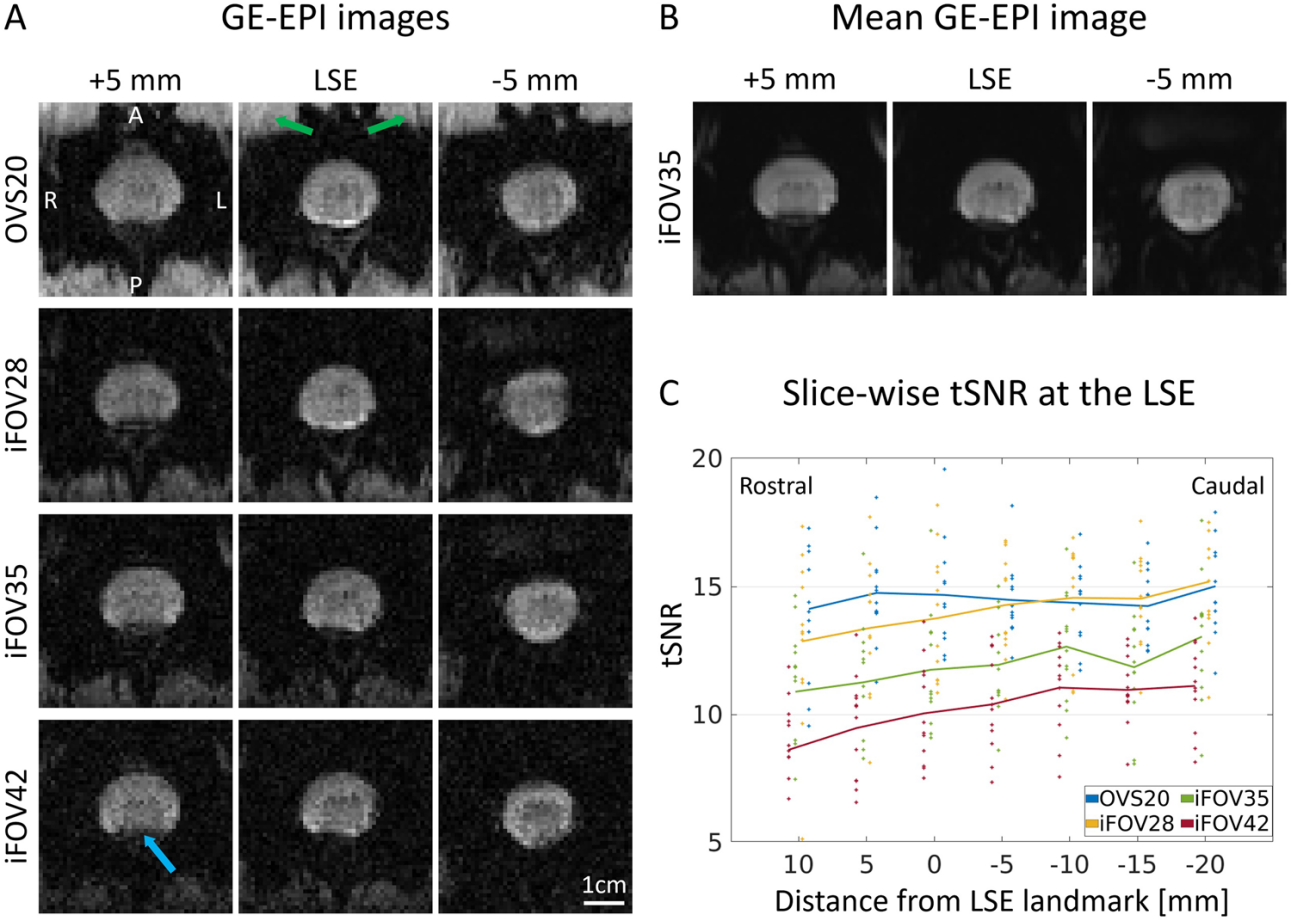
Image quality and temporal signal-to-noise ratio. (A) Example slices from each gradient-echo echo planar imaging (GE-EPI) sequence acquired in the same participant. The three displayed slices represent the slice labeled as the lumbosacral enlargement (LSE) landmark (defined inSection 2.5.5) as well as the slice above (+5 mm) and below (-5 mm). For the OVS20 sequence, notice the fold-over artifact at the anterior edge of the image (indicated by the green arrows), resulting from imperfect saturation bands. Notice the higher smoothness of the images acquired with OVS20 and iFOV28 compared with those acquired with iFOV35 and iFOV42, which results from the application of 6/8 phase partial Fourier. The dorsal part of the spinal cord is occasionally affected by susceptibility artifacts (indicated by the blue arrow). (B) Mean image (averaged across all volumes) for the iFOV35 sequence. (C) Temporal signal-to-noise ratio (tSNR) in the spinal cord for each slice and GE-EPI sequence, computed on the rest runs. Values represent group mean ± standard deviation (n = 12). The individual axial slice stacks were aligned at the LSE landmark, that is, the slice with the largest spinal cord cross-sectional area. A positive distance indicates a rostral direction from the LSE landmark. Displayed are slices where the full sample size (n = 12) was available.

The tSNR values differed significantly between sequences (p < 0.001) (Fig. 3C). Sequences with shorter TE had higher tSNR in the spinal cord, except for OVS20, which was not significantly different from iFOV28 (p = 0.49). For example, at the LSE landmark, the tSNR in the spinal cord was (group mean ± SD) 14.7 ± 2.1 for the OVS20, 13.8 ± 2.5 for the iFOV28, 11.8 ± 2.4 for the iFOV35, and 10.0 ± 2.0 for the iFOV42 sequence (Fig. 3C). Sequences with shorter TE also exhibited higher tSNR in the cerebrospinal fluid, with values approximately 40-50% of those observed in the spinal cord for the same TE. Given that tSNR in the spinal cord decreased more rapidly with longer TEs, the ratio between tSNR in the spinal cord and cerebrospinal fluid was higher at shorter TEs (Supplementary Table 1). Slices also had a significant effect on tSNR (p = 0.007), showing an increasing tendency in the caudal direction (Fig. 3C).

The percentage of outlier volumes ranged from 0.0% to 14.2% across task runs, although with large intersubject variability (Supplementary Table 2). None of the runs reached an outlier rate of 20% (exclusion threshold); therefore, all task runs were included in the analyses. For each sequence, the second task run had on average between 0.4 (iFOV28) and 0.8 (iFOV42) percentage points more outlier volumes than the first task run. The linear mixed-effects model and post-hoc tests did not reveal significant pairwise differences in the percentage of outlier volumes between the sequence types (Supplementary Fig. 3). The sequence acquired in the fourth position (at the end of the session) had a significantly higher percentage of outlier volumes than the second sequence (p = 0.0226), while the difference compared with the first sequence was marginally nonsignificant (p = 0.0525) (Supplementary Fig. 3).

### 3.2 Distribution of BOLD activity

#### 3.2.1 Subject-level results

**Fig. 4.**
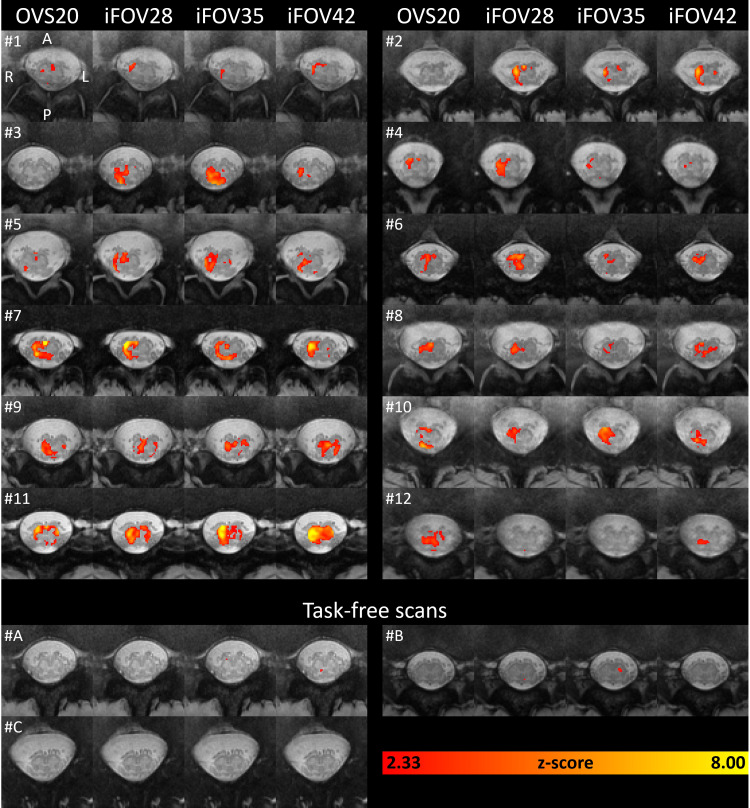
Subject-level statistical parametric maps. Parametric maps represent the z-score for the contrast task versus baseline. Parametric maps are thresholded at z > 2.33 (corresponding to uncorrected p < 0.01) and shown in the axial plane as heatmaps overlaid on the PAM50 template for each GE-EPI sequence. Three participants underwent task-free scans (i.e., the same paradigm but without performing the task). For each subject, the slice located 10 mm (2 slices) caudal to the slice with the largest spinal cord cross-sectional area is shown. Images are displayed in radiological convention.

Subject-level parametric maps displayed clusters of significant voxels in all participants when thresholded at z > 2.33 (corresponding to uncorrected p < 0.01) (Fig. 4). Across all participants, BOLD activity was predominantly lateralized on the right (ipsilateral) side of the spinal cord, with minor involvement from the left (contralateral) side. Additionally, although less pronounced, BOLD activity tended to concentrate in the ventral region of the spinal cord. Participants undergoing the task-free paradigm exhibited a minimal and spatially random pattern of activity (Fig. 4), with false positive rates of 1.40 ± 0.75% for the OVS20, 1.10 ± 0.26% for the iFOV28, 1.20 ± 0.14% for the iFOV35, and 1.15 ± 0.08% for the iFOV42 sequence when applying a threshold of z > 2.3263 (p < 0.01).

#### 3.2.2 Group-level results

**Fig. 5.**
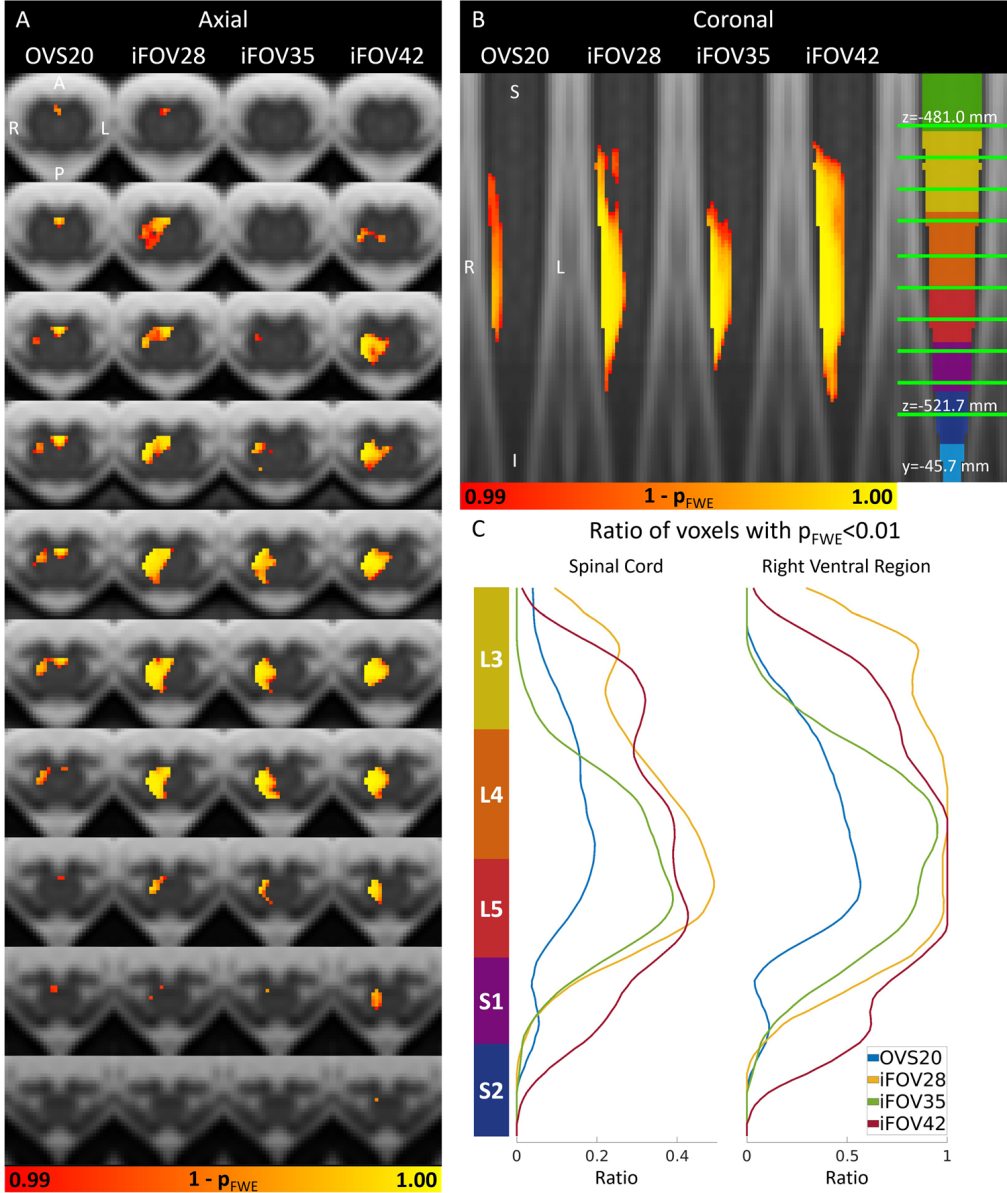
Group-level statistical parametric maps (n = 12). Parametric maps represent the family-wise error-corrected p-value (p_FWE_) for the contrast task versus baseline. Parametric maps are thresholded at p_FWE_< 0.01 and shown in both the axial (A) and coronal (B) planes as heatmaps superimposed on the PAM50 template. The axial slices are evenly spaced between z = -481.0 mm (most rostral slice, PAM50 coordinates) and z = -521.7 mm (indicated by green lines in the coronal plane). Images are displayed in radiological convention. (C) Rostrocaudal distribution of significant voxels (p_FWE_< 0.01) within the whole spinal cord and the ipsilateral (right) ventral region of the spinal cord (seeFig. 6Afor definition). Neurological levels along the rostrocaudal axis, approximated according toFrostell et al. (2016)and available in the PAM50 template, are indicated as colored segments in (B) and (C).

**Fig. 6.**
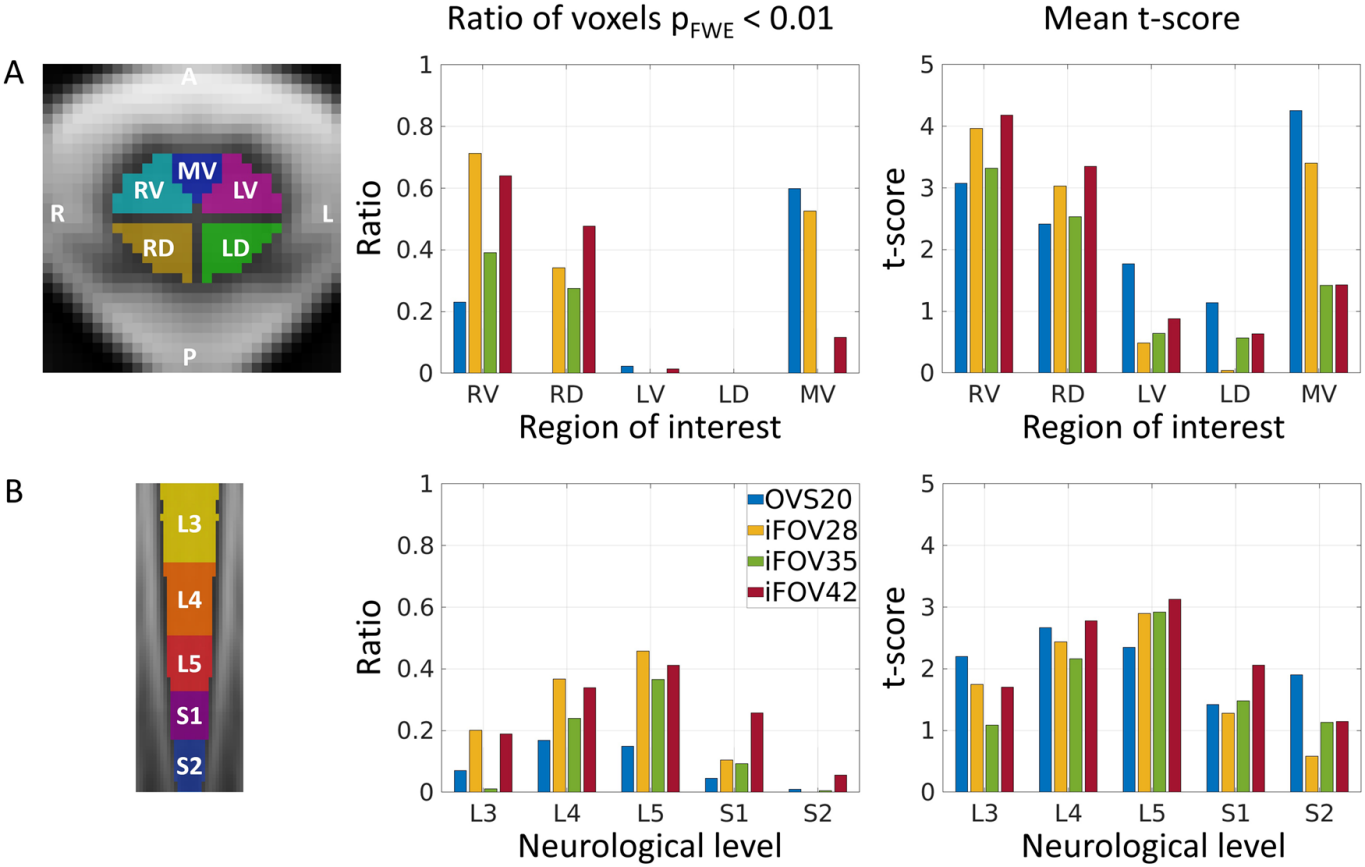
Distribution of BOLD activity at group level (n = 12). Ratio of significant voxels (p_FWE_< 0.01) and mean*t*-score within (A) each region of interest over neurological levels L3-S2 and (B) each neurological level over the whole spinal cord cross-section. The regions of interest are shown for a single slice and included the right ventral (RV), right dorsal (RD), left ventral (LV), left dorsal (LD), and medioventral (MV) spinal cord. Neurological levels along the rostrocaudal axis, approximated according toFrostell et al. (2016)and available in the PAM50 template, are indicated as colored segments in (B).

Group-level parametric maps are presented inFigure 5A and B, while plots illustrating the ratio of significant (suprathreshold) voxels and mean*t*-scores across ROIs and neurological levels are displayed inFigures 5Cand6. Numerical values are provided inTable 2. For each GE-EPI sequence, a cluster of activity was found in the right (ipsilateral) ventral region of the spinal cord, which also extended into the right dorsal region (Fig. 5A). This was also evident in the high ratio of significant voxels (p_FWE_< 0.01) and high*t*-score in the right ventral and right dorsal ROIs (Fig. 6A). At the same threshold, little to no activity was observed in the left (contralateral) ventral and dorsal ROIs. Along the rostrocaudal axis, significant voxels were detected between L3 and S2, with the highest ratio of significant voxels and highest mean*t*-score at L5 (Figs. 5Cand6B).

**Table 2.** Group-level results.

		Region of interest					Neurological level				
	Sequence	RV	RD	LV	LD	MV	L3	L4	L5	S1	S2
***t*** **-Score (mean)**	OVS20	3.08	2.41	1.77	1.14	4.25	2.20	2.66	2.35	1.42	1.91
	iFOV28	3.97	3.03	0.49	0.04	3.40	1.75	2.44	2.90	1.28	0.58
	iFOV35	3.32	2.53	0.64	0.57	1.42	1.09	2.16	2.92	1.48	1.13
	iFOV42	4.18	3.35	0.88	0.64	1.43	1.70	2.78	3.13	2.06	1.14
** p _FWE_ < 0.05 (ratio) **	OVS20	0.62	0.35	0.11	0.02	0.83	0.26	0.42	0.38	0.22	0.12
	iFOV28	0.89	0.62	0.01	0.00	0.68	0.37	0.46	0.52	0.27	0.06
	iFOV35	0.66	0.45	0.00	0.01	0.06	0.10	0.35	0.47	0.24	0.05
	iFOV42	0.84	0.73	0.07	0.00	0.27	0.33	0.46	0.53	0.39	0.14
** p _FWE_ < 0.01 (ratio) **	OVS20	0.23	0.00	0.02	0.00	0.60	0.07	0.17	0.15	0.05	0.01
	iFOV28	0.71	0.34	0.00	0.00	0.53	0.20	0.37	0.46	0.10	0.00
	iFOV35	0.39	0.28	0.00	0.00	0.00	0.01	0.24	0.37	0.09	0.01
	iFOV42	0.64	0.48	0.01	0.00	0.12	0.19	0.34	0.41	0.26	0.06
** p _FWE_ < 0.001 (ratio) **	OVS20	0.00	0.00	0.00	0.00	0.11	0.01	0.03	0.00	0.00	0.00
	iFOV28	0.23	0.11	0.00	0.00	0.24	0.02	0.18	0.24	0.01	0.00
	iFOV35	0.13	0.05	0.00	0.00	0.00	0.00	0.05	0.16	0.01	0.00
	iFOV42	0.29	0.12	0.00	0.00	0.00	0.03	0.14	0.24	0.03	0.00

Mean*t*-score and ratio of voxels with p_FWE_< 0.05, 0.01, and 0.001 within each region of interest (over neurological levels L3-S2) and each neurological level (over the whole spinal cord cross-section).

FWE, family-wise error corrected; LD, left dorsal; LV, left ventral; MV, medioventral; RD, right dorsal; RV, right ventral.

We observed differences in the level and extent across the GE-EPI sequences. The ratio of significant voxels (p_FWE_< 0.01) and the mean*t*-score in both the right ventral and dorsal ROIs tended to increase with longer TE (Fig. 6A). Notably, the OVS20 sequence showed only a minimal number of significant voxels in the right dorsal ROI. Sequences with shorter TE (20 and 28 ms) displayed a cluster of activity in the medioventral ROI, which was barely present at longer TE (35 and 42 ms) (Fig. 5A,Fig. 6A).

### 3.3 BOLD effect size

The BOLD effect size showed an increasing trend with increasing TE, with values (group mean ± SD) of 0.34 ± 0.21% for the OVS20, 0.33 ± 0.20% for the iFOV28, 0.39 ± 0.20% for the iFOV35, and 0.58 ± 0.26% for the iFOV42 sequence.

### 3.4 Reproducibility

The Dice coefficients, calculated between the two binarized statistical maps generated using solely data from either run 1 or run 2, are displayed inTable 3. Generally, the Dice coefficients were lower when applying a more conservative statistical threshold for binarization. For each statistical threshold, the iFOV42 sequence exhibited the highest Dice coefficient.

**Table 3.** Split-half reliability results.

	Dice coefficient		
Sequence	p _FWE_ < 0.05	p _FWE_ < 0.01	p _FWE_ < 0.001
OVS20	0.47	0.55	0.00
iFOV28	0.71	0.69	0.50
iFOV35	0.59	0.47	0.00
iFOV42	0.76	0.69	0.59

Dice coefficients between the group-level statistical parametric maps, computed from the first and second runs, respectively, and binarized at p_FWE_< 0.05, 0.01, and 0.001.

FWE, family-wise error corrected.

## 4 Discussion

Task-related BOLD activity associated with ankle movement is detected in the locations expected from neuroanatomical considerations, which demonstrates the feasibility and validity of lumbosacral BOLD fMRI by using reduced FOV single-shot gradient-echo echo planar imaging (GE-EPI) sequences. While all investigated GE-EPI sequences detected BOLD activity, the one with the longest echo time (TE = 42 ms) yielded the highest BOLD effect size and the highest split-half reliability. Sequences with TE below 30 ms were found to be susceptible to spatially unspecific BOLD activity due to large vein effects. The overall small BOLD effect size of approximately 0.5% underscores the necessity for effective denoising. Further research aimed at optimizing spinal cord fMRI has the potential to improve the sensitivity and robustness of lumbosacral fMRI.

### 4.1 Lateralized BOLD activity in response to unilateral lower extremity motor task

The primary cluster of BOLD activity, induced by unilateral (right-sided) ankle plantar and dorsiflexion, was extremely lateralized on the ipsilateral (right) side, aligning with our current understanding of spinal cord organization. Minimal to no activity was detected on the contralateral (left) side. Lateralization was also observed in previous fMRI studies involving upper extremity motor tasks, such as wrist flexion (Weber et al., 2016b), hand grasping (Braaß et al., 2023;Hemmerling et al., 2023), wrist extension, wrist abduction, and finger abduction (Kinany et al., 2019).

In the cross-section, the BOLD activity was centered at the ipsilateral ventral spinal cord. The spatial extent of this cluster depended on the statistical threshold applied. At a conservative threshold (p_FWE_< 0.001), the cluster was primarily localized within the ipsilateral ventral gray matter (Supplementary Fig. 4B). This distribution aligns with the currently established neuroanatomy, as the corticospinal tracts, responsible for transmitting voluntary motor commands from supraspinal centers, project to lower motor neurons located in the ventral gray matter horns, as well as to interneurons located in the intermediate zone and ventral gray matter horns (Kandel et al., 2021). The sensory feedback during voluntary movements involves the proprioceptive pathways and is necessary for correct ankle placement and timing (Akay et al., 2014;Mayer et al., 2018;Takeoka et al., 2014). The afferent Ia fibers from proprioceptors synapse with interneurons in the intermediate zone and with lower motor neurons in the ventral gray matter horns (Kandel et al., 2021;Shadrach et al., 2021). Therefore, we argue that the observed BOLD activity in the ipsilateral ventral spinal cord is likely related to the activation of both lower motor neurons and interneurons.

At more liberal thresholds (p_FWE_< 0.01,Fig. 5, and p_FWE_< 0.05,Supplementary Fig. 4A), BOLD activity extended further into the ipsilateral dorsal spinal cord. This mirrors previous reports reviewed inLandelle et al. (2021), where two-thirds of the reviewed studies utilizing fMRI in the cervical cord with upper motor tasks also reported activity in the ipsilateral dorsal horn. As mentioned above, interneurons in the intermediate zone receive synaptic input from both the afferent Ia fibers from proprioceptors and the efferent corticospinal fibers. Since we did not have a separate ROI for the intermediate zone, which is instead split between the ventral and dorsal ROIs, some of the activity from the interneurons located in the intermediate zone likely contributes to the activity in the dorsal ROI, especially in its ventral aspects. Other factors could also contribute to the dorsal spreading of the observed BOLD activity, including the relatively wide point spread function of the BOLD signal for GE-EPI sequences at 3T (Uludaǧ et al., 2009), limited spatial resolution, as well as interpolation and smoothing during preprocessing.

BOLD activity is not confined to the gray matter but extends into the surrounding white matter, as particularly evident at a liberal threshold (p_FWE_< 0.05,Supplementary Fig. 4A). On one hand, the BOLD contrast arises from the venous capillaries and other draining vessels surrounding the activated neurons (Keilholz et al., 2006;Kennerley et al., 2010), limiting the spatial specificity and representing an inherent limitation of the technique. On the other hand, smoothing the images introduces further blurring of the activity patterns.

The motor task involved ankle dorsiflexion and plantar flexion, which are executed by myotomes L4 and S1, respectively. Indeed, all sequences detected activity ranging from L3 to S2, with a single peak observed at L5. We argue that the absence of two distinct peaks, reflecting ankle dorsiflexion and plantar flexion, can be attributed to the intersubject variation in nerve root muscle innervation along with the limited rostrocaudal resolution (5 mm slice thickness), which blurs the two peaks into a single one at group level. Furthermore, studies have demonstrated that the involved muscles can be activated from various nerve roots (Hashimoto et al., 2023;Lorach et al., 2023;Phillips & Park, 1991).

### 4.2 Group activity maps are influenced by the echo time

Although BOLD activities were detected in the ipsilateral ventral gray matter horn (the expected location) using each of the four GE-EPI sequences (with TE of 20, 28, 35, and 42 ms), differences in the level and spatial pattern of these activities were also observed. At TE values below 30 ms (sequences OVS20 and iFOV28), a focal activity was identified in the medioventral region of the spinal cord. Notably, at the shortest investigated TE (20 ms), this cluster exhibited even stronger activity than the one in the ipsilateral ventral region and was the only one to survive a more conservative threshold of p_FWE_< 0.001. We argue that this cluster results from task-induced susceptibility changes in large draining vessels, indirectly reflecting neural activity in the ventral gray matter horns. First, the location of the cluster corresponds to that of the medial sulcal vein, the major venous vessel draining the ventral gray matter horns (Thron, 2016). Second, task-free controls did not exhibit similar activities. In fact, previous studies have highlighted the role of large draining vessels in contributing to the BOLD contrast (Engel et al., 1997;Lai et al., 1993;Uludaǧ et al., 2009). Consistent with our findings, the contribution of large veins to the BOLD contrast has been reported to be higher at shorter TE values (Triantafyllou et al., 2011).

We also observed an increase in the BOLD effect size within the ipsilateral ventral region with longer echo times, ranging from 0.35% (TE = 20 ms) to 0.58% (TE = 42 ms). Similarly, the ratio of significant voxels and the mean*t*-score within the same region tended to increase with longer echo times. We note, however, that these latter changes were not monotonic, as iFOV35 yielded lower values than iFOV28. We argue that the values obtained with a TE of 28 ms are inflated due to the strong activity from the neighboring medioventral region (large vein effects).

In theory, the BOLD effect size in a given voxel exhibits a broad peak, reaching its maximum when the TE approximately corresponds to the transversal relaxation time (T_2_*) within that voxel. Although this relationship often does not hold in practice due to the presence of thermal and physiological artifacts (Fera et al., 2004), the broad peak ensures that BOLD contrast is detectable within a relatively wider range of TE values around the optimal value (Menon et al., 1993;van der Zwaag et al., 2009). While we are not aware of any reports on T_2_* values in the lumbosacral cord, T_2_* was measured to be 41.3 ± 5.6 ms in the gray matter of the cervical spinal cord at 3T (Barry & Smith, 2019). This measurement aligns with our observation of the highest BOLD effect size occurring at a TE of 42 ms. It is worth noting that T_2_* values measured in the cervical cord are shorter compared with those in the brain cortex (Barry & Smith, 2019), emphasizing that a TE optimized for the brain might not be optimal for the spinal cord.

Establishing a single optimal TE is challenging in practice, as the local T_2_* depends on various factors, including shimming quality and sequence parameters such as voxel size. Moreover, there is a significant spatial variability in T_2_* in both the brain and spinal cord due to differences in magnetic susceptibility among surrounding tissues (Barry & Smith, 2019). We observed the highest*t*-scores and BOLD effect size within the ipsilateral ventral region, as well as the highest split-half reliability at a TE of 42 ms. Based on these results, we suggest that among the investigated TE values, a TE of 42 ms is optimal for detecting spatially specific BOLD activity when using a GE-EPI sequence with second-order shimming and a resolution of 1 x 1 x 5 mm^3^. This TE is longer than those typically used in recent spinal cord BOLD fMRI studies. We note that a longer TE might detect even higher BOLD activity. However, an increase in TE also prolongs the repetition time (TR), resulting in a reduction in the number of acquired volumes within the same imaging time, which, in turn, reduces the power of the fMRI experiment (Murphy et al., 2007).

### 4.3 Considerations for image acquisition

Several early spinal cord fMRI studies utilized the SEEP contrast to detect task-related correlates of neural activity (Ghazni et al., 2010;Kornelsen & Stroman, 2004;Ng et al., 2006); however, the SEEP contrast has been the subject of debate (Jochimsen et al., 2005). Additionally, the GE-EPI sequence, which targets the BOLD contrast, demonstrated higher sensitivity, spatial specificity, and reproducibility compared with the turbo spin-echo fMRI sequence, which targets the SEEP contrast (Bouwman et al., 2008;Jochimsen et al., 2005).

The reduced FOV single-shot GE-EPI sequences utilized in this study were adapted from sequences employed in previous studies (Kinany et al., 2019,^2022^;Weber et al., 2016a,2016b,^2020^), where the sequence’s sensitivity to detect BOLD activity in the spinal cord was established. The in-plane resolution and matrix size were also comparable with the spinal cord diffusion MRI sequence in the generic cervical MRI protocol (Cohen-Adad et al., 2021a).

We argue that tSNR serves as a valuable tool for evaluating the quality of shimming and image processing steps, and for estimating the statistical power of the fMRI experiment (Murphy et al., 2007). However, the observation that the sequence with the lowest tSNR (iFOV42) yielded the highest BOLD effect size clearly demonstrates that tSNR alone is inadequate for assessing the sequences’ sensitivity to BOLD signal. For example, caution is necessary when comparing sequences with different partial Fourier acceleration factors. The application of partial Fourier introduces smoothing in the image, which increases the*apparent*tSNR but reduces the intrinsic tSNR. Therefore, the observed increases in tSNR when using shorter TE are attributed not only to T_2_* decay but also to the application of partial Fourier. Note that for the iFOV sequences, the shorter TE also led to a shorter TR (with adjusted flip angle), which counteracts the tSNR increase; however, this effect appeared to be minor. Despite the shorter TE and longer TR, the OVS20 sequence yielded similar results to iFOV28, suggesting that, given the same TE and TR, inner field-of-view excitation would result in higher tSNR. This may be due to the imperfect saturation band, causing aliasing in the ventral part of the image (Fig. 3A), which might generate ghosts overlaying the spinal cord.

While not statistically significant, sequences with longer TE tended to show an increased number of outlier volumes. Considering the counterbalanced order of sequences, this trend might not be attributed to a higher level of motion toward the end of the imaging session but rather to the lower tSNR and higher artifact level associated with longer TE. Furthermore, the smoother appearance of the images when applying partial Fourier might reduce image variation and hence the number of detected outliers.

### 4.4 Limitations and future directions

Automatic segmentation techniques for the spinal cord in EPI-based fMRI data are yet to be developed and validated, leaving manual segmentation as the current standard procedure. This is a recognized issue within the research community and an ongoing joint effort is underway (Bautin & Cohen-Adad, 2021). While manual segmentation leads to intra- and inter-rater variability in the processing pipeline, it has been demonstrated that this variability does not introduce systematic bias into the group-level fMRI activity maps (Hoggarth et al., 2022).

A challenge specific to lumbosacral cord imaging is the difficulty of identifying neurological levels in vivo, given the mismatch between vertebral and neurological levels in the lumbosacral cord (Canbay et al., 2014), which precludes the use of vertebral levels as neuroanatomical landmarks. While the two-label normalization approach (Section 2.5.5) ensured the alignment of the center of the lumbosacral enlargement and the tip of the spinal cord across participants, this approach does not ensure the alignment of neurological levels in between these two landmarks. However, even if we could identify neurological levels, one must be aware of the considerable between-subject variation in the distribution of the targeted neuronal populations across neurological levels. For example, the rostrocaudal location of two muscle projectomes was even found to be inverted in an individual with spinal cord injury (Rowald et al., 2022).

We used the canonical HRF implemented in FSL, which was originally devised for the brain, for modeling the BOLD-related signal changes in the general linear model. However, the HRF might be different between the brain and in the spinal cord but even among different parts of the spinal cord (Giulietti et al., 2008), compromising the use of a single HRF across the entire FOV. While we addressed variations from the canonical HRF by using FLOBS in the general linear model, residual differences might still affect the group-level analyses (Handwerker et al., 2004).

Although visual cues were given to participants to initiate movements, we did not measure the muscle forces associated with ankle dorsi- and plantar flexion due to the unavailability of MRI-compatible dynamometers for lower extremity tasks. Therefore, drawing associations between muscle force and BOLD activity was not feasible.

In this study, we compared sequences with varying echo times in terms of their ability to detect task-related activity. Comparison of these sequences in terms of their ability to detect resting-state functional connectivity will be the focus of future investigations. Notably, a previous study performed in the cervical cord has observed differences in dynamic functional connectivity patterns when employing different sequence parameters (Kinany et al., 2022).

Finally, it is important to note the considerable intersubject variability in both the level and extent of BOLD activity (Fig. 4). We observed that participants with a higher number of outlier volumes tended to have fewer significant voxels (Supplementary Fig. 5). Interestingly, individuals who exhibited many outliers were mostly those with the least MRI experience. This suggests that those with more MRI experience might find it easier to relax and avoid motion artifacts. Nevertheless, additional sources of intersubject variability remain unknown and warrant further investigation. Moreover, a more in-depth examination of lumbosacral fMRI within a test-retest setting is necessary.

## 5 Conclusions

We demonstrated the feasibility of detecting motor-evoked BOLD activity within the lumbosacral cord using BOLD fMRI in combination with a block-design lower extremity motor task. The detected activity is in line with the expected location from neuroanatomical considerations. We further highlight the importance of echo time for the BOLD effect size and reliability in the lumbosacral cord. Functional MRI in the lumbosacral cord has significant implications for assessing motor function and its alterations in disease or after spinal cord injury.

## Supplementary Material

Supplementary Material

## Data Availability

The data can be made available upon request; however, due to ongoing analyses, they are not publicly accessible at this time. The code to process and analyze the images can be accessed atgithub.com/NeuroimagingBalgrist/LumbosacralfMRI.
