# Supplementary material for: Functional magnetic resonance imaging of the lumbosacral cord during a lower extremity motor task
Source: Imaging Neurosci (Camb). 2024 Jul 15;2:imag-2-00227. doi: 10.1162/imag_a_00227 (PMC12272214; doi:10.1162/imag_a_00227)
Supplement: Supplementary Material [file imag_a_00227-supp.pdf]

## Supplementary material

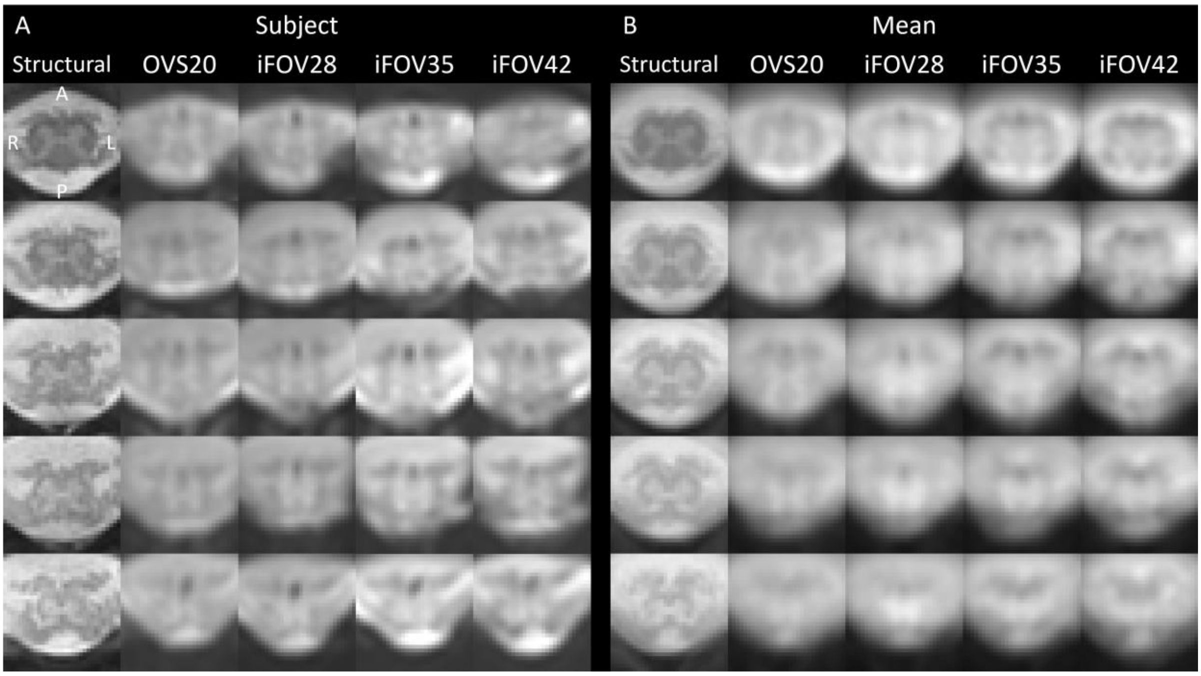


**Supplementary Figure 1.** Normalized images in the PAM50 space. Shown are the ME-GRE structural image and the functional images for each of the four GE-EPI sequences for (A) a representative participant and (B) averaged across all participants. All images are normalized to the PAM50 space. The displayed five slices correspond to every second slice shown in Fig. 5, starting with the most superior slice.

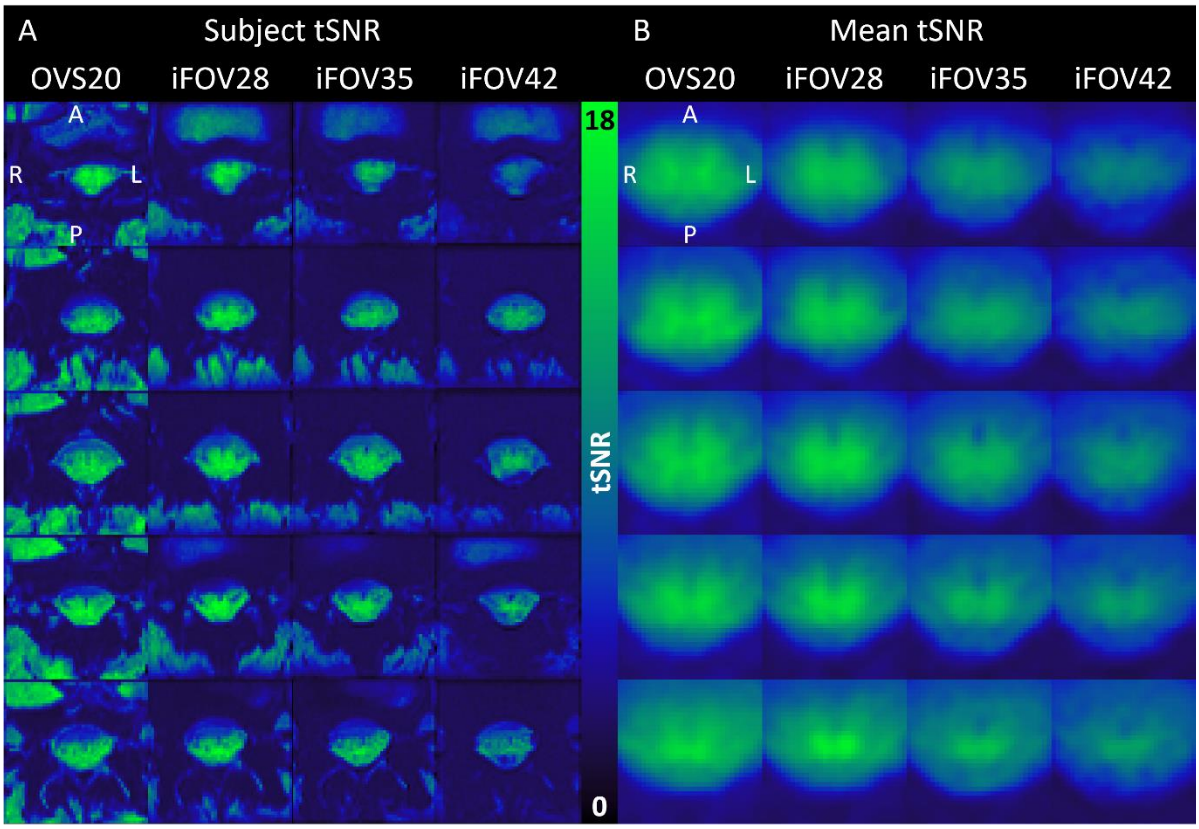


**Supplementary Figure 2.** Temporal signal-to-noise ratio (tSNR) maps computed from the functional images for each of the four GE-EPI sequences (rest runs). (A) tSNR maps for a representative participant in the native space, where the five displayed slices correspond to the slice labelled as the lumbosacral enlargement (LSE) landmark (defined in Section 2.5.5) as well as two slices above and below. (B) tSNR maps after normalization to the PAM50 space and averaging across all participants. The displayed five slices correspond to every second slice shown in Fig. 5, starting with the most superior slice. While (A) shows tSNR in a larger field of view encompassing the spinal canal, (B) displays a zoomed-in view to better appreciate the variation of tSNR within the spinal cord.

**Supplementary Table 1.** Temporal signal-to-noise ratio (group mean ± standard deviation) in the spinal cord and cerebrospinal fluid for each GE-EPI sequence, computed across seven slices where the full sample size (n=12) was available.

| **Sequence** | **tSNR** | | |
|---|---|---|---|
| | SC | CSF | SC/CSF |
| OVS20 | 14.5 ± 1.6 | 6.7 ± 1.5 | 2.6 ± 0.5 |
| iFOV28 | 14.1 ± 2.5 | 6.0 ± 1.4 | 2.3 ± 0.6 |
| iFOV35 | 11.9 ± 2.4 | 5.5 ± 1.2 | 2.2 ± 0.5 |
| iFOV42 | 10.2 ± 2.0 | 5.1 ± 1.2 | 2.1 ± 0.5 |

CSF, cerebrospinal fluid; SC, spinal cord; tSNR, temporal signal-to-noise-ratio.

**Supplementary Table 2.** Quality control. Percentage of outlier volumes detected by *fsl_motion_outliers* for each task run.

| Subject | OVS20 | | iFOV28 | | iFOV35 | | iFOV42 | |
|---|---|---|---|---|---|---|---|---|
| | Run 1 | Run 2 | Run 1 | Run 2 | Run 1 | Run 2 | Run 1 | Run 2 |
| S01 | 10.7 | 9.2 | 4.0 | 8.7 | 6.7 | 9.9 | 6.3 | 9.1 |
| S02 | 3.9 | 6.8 | 5.0 | 1.6 | 5.4 | 5.6 | 8.2 | 5.8 |
| S03 | 1.5 | 2.9 | 2.8 | 1.6 | 6.0 | 10.1 | 1.6 | 1.6 |
| S04 | 1.5 | 1.7 | 1.2 | 1.0 | 2.4 | 1.1 | 0.9 | 2.8 |
| S05 | 1.9 | 0.7 | 0.4 | 0.4 | 0.6 | 1.3 | 0.0 | 0.2 |
| S06 | 1.9 | 8.8 | 2.4 | 3.4 | 3.2 | 2.8 | 3.3 | 1.6 |
| S07 | 1.2 | 0.7 | 0.2 | 0.4 | 0.0 | 1.3 | 0.2 | 0.5 |
| S08 | 3.4 | 3.2 | 1.4 | 2.8 | 3.6 | 4.7 | 0.2 | 0.5 |
| S09 | 4.9 | 3.6 | 3.2 | 4.8 | 4.1 | 3.2 | 4.4 | 6.8 |
| S10 | 1.5 | 3.6 | 1.2 | 2.2 | 6.9 | 6.2 | 9.3 | 14.2 |
| S11 | 1.7 | 0.7 | 1.2 | 1.2 | 1.3 | 1.7 | 1.9 | 2.6 |
| S12 | 0.7 | 0.5 | 3.2 | 2.8 | 4.1 | 4.1 | 1.2 | 1.4 |
| Mean | 2.9 | 3.5 | 2.2 | 2.6 | 3.7 | 4.3 | 3.1 | 3.9 |
| SD | 2.6 | 3.0 | 1.4 | 2.2 | 2.2 | 3.0 | 3.1 | 4.1 |

SD, standard deviation.

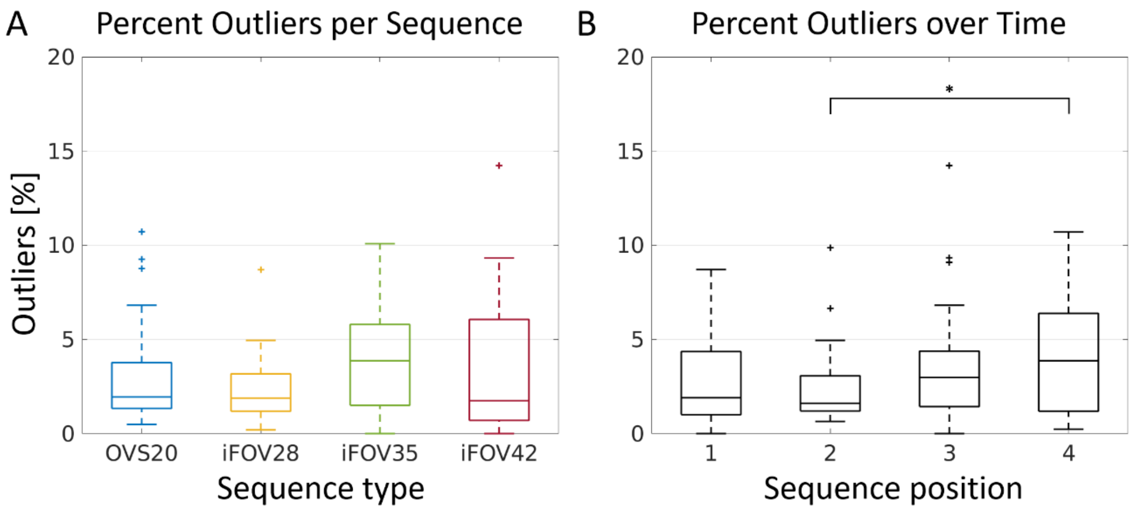


**Supplementary Figure 3.** Quality control. The boxplots illustrate the percentage of outlier volumes detected by *fsl_motion_outliers* (A) across sequences, irrespective of their relative position within the experiment, and (B) over time, considering their relative position within the experiment regardless of the sequence they belong to. Note that each sequence comprises two runs; here, the percentage of outlier volumes was computed across both runs. Values for each run are provided in Supplementary Table S1. No significant pairwise differences were found across sequence types. However, the sequence acquired in the fourth position (last sequence) had a significantly higher percentage of outlier volumes compared to the second sequence (indicated by *, p=0.0226, linear mixed-effects model with Tukey's correction).

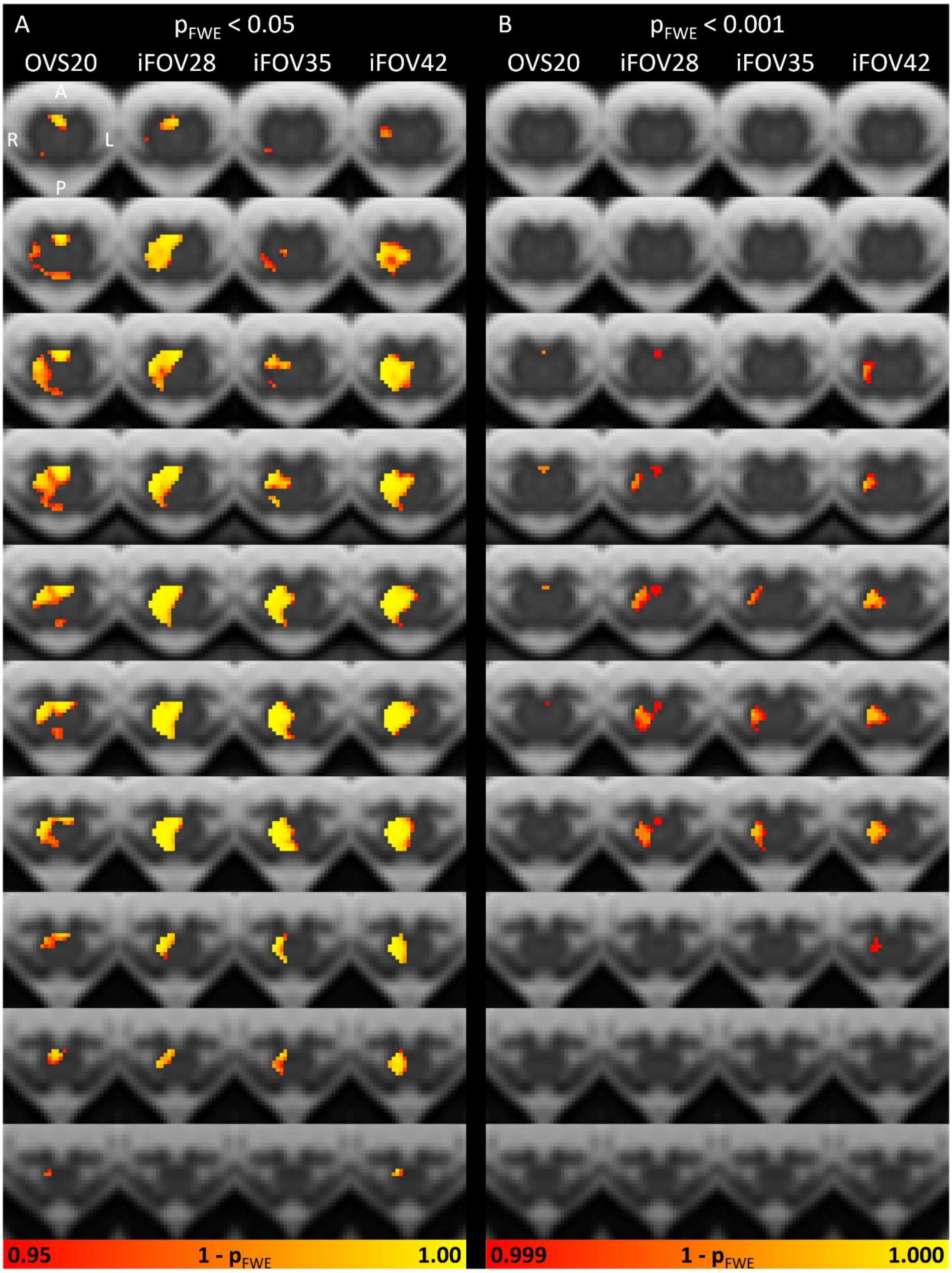


**Supplementary Figure 4.** Group-level statistical parametric maps (n=12) with alternative thresholds ($p_{FWE}$<0.05 and $p_{FWE}$<0.001). Parametric maps represent the family-wise error corrected p-value ($p_{FWE}$) for the contrast task vs. baseline. Parametric maps are thresholded at (A) $p_{FWE}$<0.05 and (B) $p_{FWE}$<0.001 and shown in the axial plane as heatmaps overlaid on the PAM50 template for each GE-EPI sequence. The axial slices are evenly spaced

between z=-466.4 mm (most rostral slice, PAM50 coordinates) and z=-510.4 mm. Images are displayed in radiological convention.

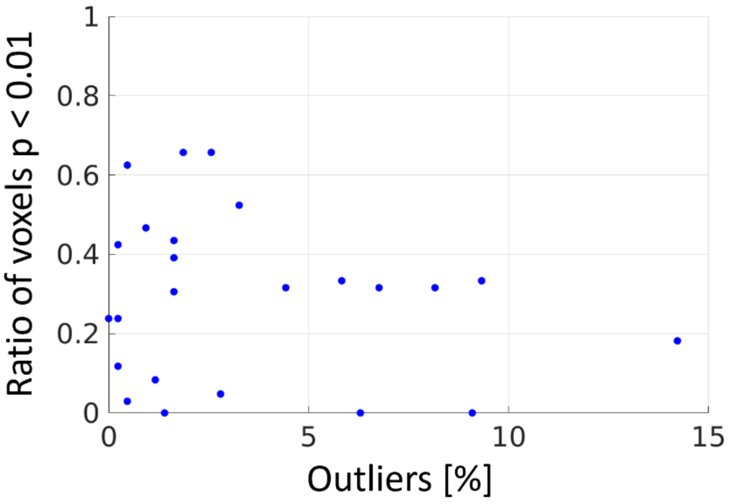


**Supplementary Figure 5.** Relationship between data quality and BOLD activity. The scatter plot displays the ratio of the significant voxels within the ipsilateral (right) ventral region (see Fig. 5A for definition) against the number of outlier volumes detected by *fsl_motion_outliers*. Each data point represents an individual subject. Significant voxels were defined as those with a subject-level z-score above 2.33 (corresponding to an uncorrected $p < 0.01$). For each subject, the number of outlier volumes was averaged across both runs of the iFOV42 sequence (n=429 volumes in each run). While a low number of outliers (i.e., below 20) did not appear to be associated with the ratio of significant voxels, a high number of outliers tended to correspond to fewer significant voxels. While not shown here, sequences OVS20, iFOV28, and iFOV35 displayed a qualitatively similar pattern.
